# Can We Predict the Toxicity and Response to Thiopurines in Inflammatory Bowel Diseases?

**DOI:** 10.3389/fmed.2019.00279

**Published:** 2019-11-28

**Authors:** Raphael P. Luber, Sailish Honap, Georgina Cunningham, Peter M. Irving

**Affiliations:** Department of Gastroenterology, Guy's and St. Thomas' NHS Foundation Trust, London, United Kingdom

**Keywords:** thiopurines, azathioprine, 6-mercaptopurine, inflammatory bowel disease, Crohn's disease, ulcerative colitis, toxicity, response

## Abstract

Thiopurines are a cheap, effective treatment option in the management of inflammatory bowel disease (IBD). However, with the growing choice of targeted therapies available, as well as the well-documented toxicities of thiopurines, the role of thiopurines has been questioned. Nevertheless, given their inexpense in an era of spiraling healthcare costs, thiopurines remain an attractive option in the right patients. In the age of personalized medicine, being able to predict patients who will respond as well as those that will develop toxicity to a treatment is vital to tailoring therapy. This review will summarize the available literature with respect to predictors of response and toxicity to thiopurines in order to guide management in IBD. Specifically, toxicities addressed will include myelotoxicity, hepatotoxicity, pancreatitis, alopecia, gastrointestinal and flu-like symptoms, and complications associated with Epstein-Barr virus. While more work needs to be done to further our ability to predict both response to and side effects from therapies, pharmacogenomic research shows significant promise in its ability to personalize our use of thiopurines.

## Introduction

Thiopurines, including azathioprine, 6-mercaptopurine and tioguanine, are longstanding therapies within the ever-expanding inflammatory bowel disease (IBD) treatment armamentarium (1–5). They have shown themselves to be effective in the maintenance of remission in patients with IBD and have also resulted in reductions in the need for surgery, post-operative recurrence and IBD-related colorectal cancer risk. In addition, they improve pharmacokinetics of anti-tumor necrosis factor agents when used in combination with these therapies (6). Given their efficacy, oral delivery, and low cost they are frequently used as pre-biologic treatments in both Crohn's disease (CD) and ulcerative colitis (UC) and many clinicians have extensive experience with their use. However, up to 60% of patients will either respond inadequately or will develop toxicity to thiopurines (7), necessitating their cessation or treatment modification. With the continual advent of new targeted biologic therapies, the role of thiopurines in the current era is, therefore, being questioned (8).

The ability to use clinical and biologic characteristics of an individual to predict their disease course and to personalize their treatment pathway is the aim of precision medicine (9). An essential component of this goal is the ability to predict those who are more likely to respond or develop toxicity to a particular therapy, in order to improve the safety and efficacy of treatment choices. While some authorities suggest that treatment choices should not be influenced by cost, the compounding prevalence of IBD in conjunction with the increasing burden of drug costs mean that such an approach is perhaps naïve within the context of finite resources. Thus, optimizing the use of inexpensive treatments like thiopurines could have significant financial advantages to health services.

While thiopurines are still felt to have a role in the current era (8, 10), our ability to tailor their use to a population which will both tolerate them and achieve the treatment goals that our new treatment paradigms demand will determine their use in the future. This article aims to summarize the available evidence with respect to clinical, genetic, and biological predictors of response and toxicity to thiopurines.

## Predictors of Toxicity

Thiopurine use is undoubtedly hindered by the high incidence of adverse drug reactions which affect up to 25% of people who take them, resulting in drug discontinuation in 17% of patients (11). Side effects often occur in the first few months. Accordingly, the ability to predict which patients are likely to develop these potentially serious side effects would be of great use in clinical practice.

### Thiopurine-Induced Myelotoxicity

Thiopurine-induced myelotoxicity (TIM) is one of the most serious thiopurine-induced side effects and can occur at any time during treatment. In some patients this can lead to life threatening bone marrow suppression. Whilst leucopenia is the commonest hematological abnormality, thrombocytopenia, and pancytopenia can rarely occur. In a review of 66 studies, including more than 8,000 thiopurine-treated patients, the incidence rate of drug-induced myelotoxicity was 3% per patient year of treatment (12). In East Asian populations, however, the incidence of myelotoxicity can be as high as 15% (13).

The prodrug azathioprine is non-enzymatically converted to 6-mercaptopurine (6MP) and then through competing pathways is metabolized into thioguanine nucleotides (TGNs). TGNs exert their immunosuppressive effect by interfering with DNA replication of the most actively dividing cells, as well as by inducing apoptosis in activated and pre-activated T lymphocytes (14). Thiopurine-S-methyltransferase (TPMT) is an enzyme which catalyzes the methylation of 6-MP to 6-methylmercaptopurine (6-MMP), a non-therapeutic metabolite. Approximately 1 in 10 people have intermediate TPMT activity due to heterozygosity of TPMT, and 1 in 300 have TPMT deficiency, which is inherited in an autosomal recessive manner (15). In heterozygotes, TPMT^*^3A is the commonest mutant allele (85%), whilst TPMT^*^2 and TPMT^*^3C are rarer (15). TIM is strongly linked to low TPMT enzyme activity and high 6-TGN blood levels (16). Standard thiopurine dosing in heterozygous or TPMT-deficient patients leads to 6-TGN accumulation in the bone marrow and potentiates the risk of life-threatening bone marrow suppression.

TPMT phenotype testing is commonplace in clinical practice and is one of the most frequently used pharmacogenetic tests. TPMT enzyme assays can also be used alongside genotyping, which is used less commonly, to assess activity where rarer mutations may be missed on genotyping. Genetic testing is useful in patients with renal failure and reduced clearance of TPMT inhibitors, where enzyme activity can be falsely low. However, routine genotyping is not commonplace with some evidence suggesting that this may not be cost-effective compared with standard phenotyping (17, 18). TIM can also occur with normal TPMT activity necessitating regular full blood count monitoring in clinical practice to allow dose reduction or drug cessation in cases of TIM. A meta-analysis found that low TPMT is associated with TIM but not hepatotoxicity or pancreatitis (19).

Nudix hydrolase 15 (*NUDT15*) variants have also been linked to altered thiopurine metabolism and TIM (20). Mutations of *NUDT15*, which occur more frequently in the East Asian population, lead to reduced enzyme activity and TIM in a TGN-independent manner (20, 21). More recently, variants in *NUDT15* were found to be associated with increased risk of TIM among IBD patients of European ancestry (22). Furthermore, patients with mutations in both *TPMT* and *NUDT15* developed TIM faster (22). These findings highlight the importance of *NUDT15* genotyping, alongside *TPMT* phenotype, or genotype testing. This is supported by a recent systematic review and meta-analysis (23), and recently published guidelines provide suggested dosing regimens in patients with *TPMT* or *NUDT15* variants (24).

It may also be possible to predict early myelotoxicity by measuring thiopurine metabolites soon after treatment commencement. In a Dutch study, patients with 6-TGN levels of more than 213 pmol/8 × 10^8^ red blood cells (RBC) and 6-MMP levels higher than 3,525 pmol/8 × 10^8^ RBC measured after 1 week of thiopurine initiation were six times more likely to have early TIM (25).

### Thiopurine-Induced Hepatotoxicity

Thiopurine-induced hepatotoxicity (TIH) is an uncommon but important side effect of thiopurine use. Most commonly, this results in increased transaminase levels, which resolves with dose reduction or drug discontinuation. Less commonly, TIH manifests as idiosyncratic cholestasis or nodular regenerative hyperplasia. A systematic review, which included 3,485 patients, described an overall prevalence of 3.4% for TIH (26). In a pediatric cohort, TIH was found to be strongly correlated with 6-MMP levels with a 3-fold increased risk at levels >5,700 pmol/8 × 10^8^ RBC (16). In a Dutch cohort study of 270 adult patients, when TIH occurred it did so within 8 weeks in 85% of patients and was associated with elevated 6-MMP levels (27). Furthermore, in the same study, a predictive algorithm based on a week one 6-MMP level >3,615 pmol/10^8^ RBC, older age, male gender and higher BMI yielded an area under the curve of 0.83 (95% CI: 0.75–0.91) for hepatotoxicity risk. Another study found elevated 6-MMP levels in those with TIH but sensitivity and specificity were poor (28). These studies highlight that whilst 6-MMP levels are associated with TIH, intervention should be reserved for those in whom the high 6-MMP levels are associated with abnormal liver function tests.

Nodular regenerative hyperplasia is a condition characterized by diffuse nodulation of the hepatic parenchyma, leading to portal hypertension. Although its natural history is not clearly understood, it can occur in patients treated with purine analogs, particularly tioguanine (29). Studies have shown that this is more likely to occur in male patients with a stricturing small bowel disease phenotype (30–32). Although rare, regular monitoring of blood tests is necessary, particularly for the gradual onset of thrombocytopenia signaling portal hypertension.

Although there is no validated genetic predictor of TIH, there is a worldwide collaborative effort to achieve this aim. This includes the Helmsley IBD Exome Sequencing Program (33), and the Predicting Serious Side Effects in Gastroenterology (PRED4), conducted by the UK IBD Genetics Consortium.

### Thiopurine-Induced Pancreatitis

Pancreatitis occurs in <5% of patients treated with azathioprine or mercaptopurine and often occurs in the first month of treatment (11, 34, 35). Reinstating therapy upon recovery leads to recurrent pancreatitis, so indefinite drug withdrawal is required although a switch to tioguanine may be considered (34, 36). Thiopurine-induced pancreatitis (TIP) is an idiosyncratic drug reaction and the pathophysiology is unknown. Interestingly, and for unclear reasons, patients treated with thiopurines for IBD have a greater incidence of TIP compared to those treated for other diseases (37). However, TIP is almost always mild in IBD patients and generally responds rapidly to drug withdrawal (38). Smoking has been found to be a strong risk factor in TIP (39), along with having CD (11, 38).

Two genome wide association studies of patients with TIP found a link to the class II HLA region, with the most significant associations identified being at rs2647087 (40, 41). Patients heterozygous at rs2647087 have a 9% risk of developing TIP and homozygotes have a 17% risk (40) although tests to predict risk of TIP are not yet commonplace in clinical practice. Approximately 76 patients need to be genotyped for rs2647087 to prevent one case of pancreatitis, and given that most cases of TIP run a benign course, there is an argument that screening may not be a cost-effective strategy.

### Thiopurine-Induced Alopecia

Alopecia secondary to thiopurine use is a rare, dose-related adverse event, with an incidence of 1.5% in patients of Asian descent (13). Whilst clearly not life-threatening, alopecia can have profound psychological effects and increases the risk of non-compliance with therapy. Studies have shown that the *NUDT15* variants are associated with risk of thiopurine-induced alopecia (42, 43). Therefore, dose reduction in heterozygotes and thiopurine avoidance in homozygotes can mitigate and avoid both TIM and alopecia in this cohort.

### Gastrointestinal Toxicity and Flu-Like Illness

The most common but least serious adverse effects of thiopurines are gastrointestinal disturbances (nausea, vomiting, abdominal pain) and flu-like symptoms (malaise, fever, myalgia), which are responsible for drug discontinuation in many patients. The flu-like symptoms are likely to be immune-mediated and tend to occur shortly after starting treatment. It is not clear if the reactions are dose-dependent or idiosyncratic. A prospective evaluation of azathioprine-treated IBD patients found that *TPMT* heterozygosity strongly predicted GI adverse effects (37% heterozygous vs. 7% wild-type *TPMT, P* < 0.001) (44).

Switching treatment to 6MP may be one way to curb some of these side effects. An observational study and systematic review demonstrated 60% of patients intolerant of azathioprine were able to tolerate 6MP (45). In those ceasing 6MP due to further adverse effects, 59% experienced the same side effect as they had with azathioprine.

### Serious Complications Associated With Epstein-Barr Virus (EBV)

The association between thiopurine use and EBV-driven B-cell lymphoma has been understood for many years. A roughly four-fold increase in risk over background has been identified across several studies (46) and, thus, the greatest absolute risk is in those with the highest background risk, i.e., the elderly.

In addition, severe and potentially fatal EBV primary infections and post infectious lymphoproliferative disorders have also been associated with thiopurine use (46–48). This has prompted some to advocate for pre-treatment EBV serology testing and avoidance of thiopurines, if possible, in EBV seronegative individuals (48). In the CESAME study (Cancers Et Surrisque Associé aux Maladies inflammatoires intestinales En France), a low incidence of 0.1 per 1,000 patient years of postmononucleosis lymphomas was observed overall, rising to 3 per 1,000 patient years when considering young males, seronegative for EBV (46). In addition, in a pediatric population of 5,766 participants, there were 5 cases of haemophagocytic lymphohistiocytosis (HLH), all exposed to thiopurines, equating to an incidence of 0.2 per 1,000 patient years (49).

Despite the majority of pediatric patients being EBV seronegative at initiation of thiopurines (50), the incidence of HLH is low. Furthermore, as EBV is not the sole trigger of serious infectious complications like HLH (47), some argue against routine pre-thiopurine EBV testing (51, 52). Nevertheless, given the potentially severe, albeit rare, consequences of primary EBV infections or post infectious lymphoproliferative disorders in patients on thiopurines, coupled with the increasing availability of therapeutic alternatives, we carefully balance the risk and benefit of thiopurine use in EBV negative patients, but do not avoid its use completely.

## Predictors of Response to Thiopurines

The ability to predict who will respond to thiopurine therapy and to maximize likelihood of response earlier in the disease course would enable clinicians to tailor therapy sooner, with the aim of altering the natural history of the disease (53). Heterogeneity in definitions of response, as well as the tenuous relationship between clinical response and mucosal activity make interpretation of the literature challenging with respect to prediction of response. While thiopurine metabolite monitoring enables personalized dosing, it obviously relies on patients having already commenced the therapy; pre-treatment predictors are the ideal.

### Clinical Predictors

The relatively small numbers of patients in azathioprine efficacy studies has limited our ability to identify clinical predictive factors of response (3, 54). As such, clinical predictive factors have thus far not been incorporated into clinical practice in a significant way. A number of retrospective studies have identified factors that may predict response, or lack of it, although their results must be interpreted with caution.

Some of the largest studies addressing predictive factors of thiopurine response have yielded conflicting results in terms of disease type (CD vs. UC) and location. In a single center review of 622 patients, remission rates in those who completed 6 months of azathioprine were highest in UC patients compared to CD (87% vs. 64%, *p* = 0.0001) (55). In CD cases specifically, colonic distribution was associated with a higher rate of clinical remission compared to other distributions. This finding was mirrored in another study which found that azathioprine caused mucosal healing in 70% of patients with Crohn's colitis and 54% with ileitis (56). However, in a study of 139 IBD patients, rates of response to thiopurines were highest in patients with ileal CD (27 responders vs. 2 non-responders, *p* = 0.003) (57). No difference was found in response rate in other IBD subtypes, although numbers were small.

Body mass index (BMI) has been associated with response, surprisingly with opposite effects in UC and CD. In a large retrospective study (*n* = 1176), patients with UC with a BMI <25 had a lower flare rate after starting azathioprine than those with BMI >25, albeit only in those with disease duration <3 years (58). In CD, flare rates were similar between BMI groups, however upon azathioprine withdrawal, patients with a BMI <25 had higher flare rates than BMI >25 (58). It is theorized that adipocytes and fatty tissue may play an immunological role, involved in the physiologic and pathologic regulation of the immune system and inflammation (59). BMI, however, had no effect on thiopurine efficacy in a smaller study (57).

With regard to clinical disease activity, it has been reported that long term clinical response is improved in CD when azathioprine is commenced when patients are in remission (58). While this may reflect less severe disease, the same difference was not seen in UC. In a Korean study published in abstract form only, however, high Mayo score was associated with thiopurine treatment failure in patients with UC (HR 1.28, 95% CI 1.04–1.58, *p* = 0.023) (60). In addition, in a cohort of mixed IBD patients response was associated with shorter duration of disease at the time of commencing azathioprine than non-response (47.4 ± 6.6 months among responders, vs. 85.4 ± 14.6 in non-responders, *p* = 0.007) (57), suggesting earlier introduction of thiopurines may improve response.

In contrast to these studies, a prospective double-blind trial of patients with a recent (<8 weeks) diagnosis of CD found that rates of corticosteroid-free clinical remission at week 76 were similar in azathioprine and placebo-treated patients (61). This finding was supported by an open-label French study, in which early (<6 months) administration of azathioprine was no more effective than conventional management (62). Whilst the findings of theses studies need careful interpretation (63, 64), the early introduction of thiopurines cannot, therefore, be recommended in all patients with CD.

Ethnicity may also play a role in thiopurine metabolism and response. In an observational study of Chinese patients with UC, standard dose thiopurine (>2 mg/kg/day) was compared to low dose thiopurine therapy (<2 mg/kg/day). Cumulative relapse-free survival rates were similar between groups, however a three-fold increased risk of leucopenia was seen with standard dosing (65). This may be reflective of variations in genotypes between ethnic groups such as has been seen with variants of *NUDT15*, associated with an increased risk of leucopenia, which are more commonly found in Asian patients (22, 66).

The effect of gender has been conflicting across studies, and likely plays no role. Female gender was found to be associated with thiopurine response by some (57, 67), with the opposite found in a pediatric population (68) and no difference in another larger adult population (55).

### Thiopurine Metabolites and TPMT

6-TGN levels have been shown to correlate with efficacy (69), and levels ≥ 235 pmol/10^8^ RBC are associated with clinical response and remission in thiopurine monotherapy (16). Meta-analysis data confirm this, showing a higher rate of clinical remission in patients with a 6-TGN above this threshold compared with below (62 vs. 36%, pooled OR 3.3, 95% CI 1.7–6.3; *p* < 0.001), as well as higher TGNs in patients in clinical remission vs. active disease (53, 70). However, it must be recognized that these data are based upon studies which are small, heterogeneous and generally retrospective (71) and a prospective multicenter study of thiopurine weight-based dosed IBD patients found a poor relationship between TGN and clinical response rate, with no useful TGN cut-off determinable (72).

While a threshold of 235 pmol/10^8^ RBC may be sufficient for clinical remission, mucosal healing, increasingly recognized as a more robust, and potentially disease-modifying endpoint (73, 74), may require higher levels. A recent multicenter, international retrospective study showed that 6-TGN levels were associated with mucosal healing, and that a level of 397 pmol/10^8^ RBC was 86.7% specific but only 35.3% sensitive for mucosal healing (75). However, higher 6-TGN levels are also associated with increased rates of early or late myelotoxicity (23), particularly above 450 pmol/8 × 10^8^ RBC (76), and so a fine balance exists between response and toxicity. Interestingly, while a lower thiopurine dose may be sufficient for Asian patients, as discussed above, it is the 6-TGN and not the dose that was associated with mucosal healing in a cohort including a large proportion of Chinese patients (75).

The optimal use of thiopurine metabolite levels (6-TGN and 6-MMP) to maximize response, however, is a controversial area, with practices varying across the world (77–79). Observational data support the use of TGN monitoring in non-responding patients, with TGN-directed dose optimization eliciting a response rate of 87–90% compared to 18–33% in which treatment was not TGN directed (p<0.001 for both studies) (77, 80). In one retrospective study of 169 patients undergoing thiopurine metabolite testing, the majority (52%) had subtherapeutic 6-TGN levels and testing resulted in a change in patient treatment in 68% of patients overall and 86% of patients with active disease and sub-therapeutic levels (81).

However, prospective randomized trials of TGN monitoring vs. standard weight-based dosing in patients commencing on thiopurines for IBD have failed to show benefit (82, 83). In a study of 57 patients, rates of clinical remission in TGN-guided vs. standard weight-based dosing groups were similar at 16 and 24 weeks (82). However, it should be noted that mean 6-TGN levels in the standard group ranged between 216 and 266 pmol/10^8^ RBC. This is in contrast to real world data suggesting 50% of thiopurine-treated patients are not receiving the appropriate weight-based dose, corresponding to 40–50% being underdosed on TGN criteria (80, 81). In another prospective study of 50 patients, clinical remission rates at week 16 were higher in the TGN-based vs. weight-based dosing. However, this failed to achieve statistical significance, possibly due to underpowering (40% vs. 16%, *p* = 0.11) (83).

Thiopurine metabolite testing is also helpful when preferential 6-MMP metabolism or “shunting” occurs. This phenomenon is associated with reduced efficacy and increased side effects (84). Fortunately, it can be overcome with a reduction in dose of the thiopurine and the introduction of allopurinol (85–87). Indeed, commencing low dose thiopurine-allopurinol combination therapy at thiopurine initiation, regardless of TGNs, may achieve higher response rates and reduced side effects (88).

While measuring 6-TGN may be useful for optimizing response, it may also be useful for predicting a lack of response. In patients with active disease on thiopurine therapy, persistence despite a therapeutic 6-TGN is unlikely to result in success (80), necessitating treatment alteration.

*TPMT* activity has also been assessed with regards to its role in predicting response. In a study of 39 patients with IBD, patients with *TMPT* activity <30.5 EU/mL were more likely to have a clinical response to thiopurines than those with higher *TPMT* activity (65 vs. 29%, *p* = 0.05), independent of TGN values. In patients with *TPMT* activity <30.5 EU/mL and a therapeutic 6-TGN, 100% responded compared to 25% with higher *TPMT* activity and low 6-TGN (*p* = 0.01) (89). Others, however, have found no relationship between TPMT activity and clinical response (72). Given biologic plausibility as well as evidence that low *TMPT* activity is associated with higher 6-TGN (16), clinical response is more likely to be related to TGN than the *TPMT* activity.

### Genetic Predictors

*TPMT* polymorphisms have not been associated with response (67, 90). In a prospective multicenter trial of 783 patients randomized to either *TPMT* polymorphism pre-screening and pre-emptive dose reduction vs. standard treatment, clinical response rates did not differ (90).

A recent small pharmacogenomic study assessed the role of polymorphisms of potential genes of relevance to azathioprine metabolism on clinical response and toxicity to azathioprine in IBD. *GSTM1* deletion, a polymorphism of the gene encoding Glutathione-S-transferase, the enzyme responsible for conversion of azathioprine to 6-mercaptopurine, was significantly associated with poor response to azathioprine on multivariate analysis albeit with a wide confidence interval (OR 9.22, 95% CI 1.081–78.62, *p* = 0.042) (67).

Published in abstract form only, a predictive model for achieving corticosteroid free remission with thiopurines at 26 weeks in a pediatric cohort of mixed IBD patients showed promise. Using novel pharmacogenetic genome-wide association study-identified loci, the previously identified IBD susceptibility locus HLA-DRB-1, and clinical features including pANCA positivity, disease duration, and diagnosis of UC as opposed to CD, the model had an area under the curve for corticosteroid-free remission of 0.985 (68).

## Conclusion

Thiopurines remain an effective treatment for IBD, with their relative cost, decades of use and the ability to measure and optimize metabolites maintaining their role in the biologic era. As we strive for an era of personalized medicine and gain further experience with our expanding therapeutic choices, our ability to predict thiopurine response and toxicity, and to tailor therapy accordingly, will determine its future role. While thiopurine metabolite monitoring shows utility in those already commenced on thiopurines, pharmacogenetic testing, which already plays a significant role in preventing toxicity, shows some promise in predicting response.

## Author Contributions

RL, SH, and GC wrote and reviewed the manuscript. PI supervised and reviewed the manuscript.

### Conflict of Interest

SH has received lecture fees from Pfizer, Janssen, and Takeda. PI has received fees for speaking on behalf of or acting in an advisory capacity for AbbVie, Arena, Celgene, Ferring, Prometheus, Shire, Warner Chilcott, Takeda, MSD, Vifor Pharma, Pharmacosmos, Topivert, Genentech, Hospira, Samsung Bioepis, VH2, Janssen, Sandoz, Lilly, Roche and has received financial support for research from MSD, Pfizer, and Takeda. The remaining authors declare that the research was conducted in the absence of any commercial or financial relationships that could be construed as a potential conflict of interest.
